# An Open‐Label, Multicenter, Phase II Study of Bexarotene in Patients with Adult T‐Cell Leukemia/Lymphoma

**DOI:** 10.1111/1346-8138.17919

**Published:** 2025-08-23

**Authors:** Kentaro Yonekura, Ikko Muto, Motoi Takenaka, Jun Aoi, Taku Fujimura, Masahiro Amano, Takuro Kanekura, Takuya Miyagi, Kanami Saito, Eiji Kiyohara, Takatoshi Shimauchi, Ken Watanabe, Tomoko Miyake, Takamichi Ito, Hisashi Uhara, Kazuyasu Fujii, Akihito Sudo, Toshiki Watanabe, Keiji Iwatsuki

**Affiliations:** ^1^ Department of Dermatology Imamura General Hospital Kagoshima Japan; ^2^ Department of Dermatology Kurume University Kurume Japan; ^3^ Department of Dermatology, Graduate School of Biomedical Sciences Nagasaki University Nagasaki Japan; ^4^ Department of Dermatology and Plastic Surgery, Faculty of Life Sciences Kumamoto University Kumamoto Japan; ^5^ Department of Dermatology Tohoku University Graduate School of Medicine Sendai Japan; ^6^ Department of Dermatology, Faculty of Medicine University of Miyazaki Miyazaki Japan; ^7^ Department of Dermatology Kagoshima University School of Medicine Kagoshima Japan; ^8^ Department of Dermatology, Graduate School of Medicine University of the Ryukyus Nishihara Japan; ^9^ Department of Dermatology, Faculty of Medicine Oita University Yufu Japan; ^10^ Department of Dermatology, Graduate School of Medicine Osaka University Suita Japan; ^11^ Department of Dermatology Hamamatsu University School of Medicine Hamamatsu Japan; ^12^ Department of Dermatology Yokohama City Minato Red Cross Hospital Yokohama Japan; ^13^ Department of Dermatology, Dentistry, and Pharmaceutical Sciences Okayama University Okayama Japan; ^14^ Department of Dermatology, Graduate School of Medical Sciences Kyushu University Fukuoka Japan; ^15^ Department of Dermatology Sapporo Medical University Sapporo Japan; ^16^ Minophagen Pharmaceutical Tokyo Japan; ^17^ Department of Practical Management of Medical Information, Graduate School of Medicine St Marianna University Kawasaki Japan

**Keywords:** adult T‐cell leukemia/lymphoma, bexarotene, retionid, skin lesion, systemic therapy

## Abstract

A multicenter, open‐label, historically controlled, 2‐arm phase II study was conducted to evaluate the safety, efficacy, and pharmacokinetics of bexarotene in Japanese patients with adult T‐cell lymphoma/leukemia (ATL). The study enrolled patients with indolent ATL and skin lesions and patients with aggressive ATL who had skin relapse after achieving remission following ≥ 1 regimen of systemic chemotherapy. Patients received oral bexarotene at an initial dose of 100 mg/m^2^ (15 patients) or 300 mg/m^2^ (17 patients) once daily for 24 weeks. The 100 mg/m^2^ group included 14 patients with indolent ATL and 1 with aggressive ATL. The 300 mg/m^2^ group included 14 patients with indolent ATL and 3 with aggressive ATL. Of 12 patients in the 100 mg/m^2^ group, 6 patients achieved complete response (CR) or partial response (PR) as the best overall response (50.0%; 95% confidence interval (CI), 25.4%–74.6%). Of 17 patients in the 300 mg/m^2^ group, 12 patients achieved CR or PR (70.6%; 95% CI, 46.9%–86.7%) as estimated with the modified Severity Weighted Assessment Tool (mSWAT). Drug‐related adverse events (AEs) occurred in 15 patients (100%) in the 100 mg/m^2^ group and 16 of 17 patients (94.1%) in the 300 mg/m^2^ group. The most common AEs were hypothyroidism (75.1%) and hypertriglyceridemia (53.1%). This is the first report to evaluate the safety and efficacy of bexarotene in ATL focused on skin lesions. In both groups, the response rate exceeded the threshold defined for the study. Bexarotene might be effective in improving skin lesions in ATL of any type with good tolerability.

**Trial Registration:** This study was registered with the Japan Registry of Clinical Trials (jRCT2080224172)

## 1 Introduction

Adult T‐cell leukemia/lymphoma (ATL) is a hematological malignancy caused by human T‐cell leukemia virus type 1 (HTLV1). According to the World Health Organization (WHO) classification, ATL is classified as a mature T‐cell/natural killer (T/NK)‐cell neoplasm and defined as a peripheral T‐cell neoplasm with monoclonal integration of the HTLV‐1 provirus into tumor cell DNA [1].

ATL causes a wide variety of symptoms, including leukocytosis, skin lesions, lymphadenopathy, hypercalcemia, high serum levels of lactate dehydrogenase (LDH), hepatomegaly, splenomegaly, pleural effusion, ascites, gastrointestinal lesions, central nervous system involvement, and opportunistic infections [2].

ATL is classified into four subtypes on the basis of its clinical features: acute, lymphoma, chronic, and smoldering. The acute, lymphoma, and unfavorable chronic types are called aggressive ATL because of rapid progression and very poor prognosis [2, 3]. Unfavorable factors were defined as any one of the following: blood urea nitrogen greater than the institution's upper reference value limit, lactate dehydrogenase (LDH) greater than the institution's upper reference value limit, or serum albumin less than the institution's lower reference value limit. By contrast, smoldering ATL and favorable chronic types are called indolent ATL because the disease progresses relatively slowly with few symptoms. It has a better prognosis than aggressive ATL. However, depending on the follow‐up periods, approximately half or more patients with indolent ATL progress to the acute type, which has a poor prognosis [4, 5].

Therapeutic strategies differ by clinical subtype. Multiagent combination chemotherapy is recommended as a standard therapy for aggressive ATL [6]. For patients who respond to first‐line therapy, allogeneic hematopoietic stem cell transplantation is recommended as a treatment that is expected to achieve long‐term survival, but it has the limitation of age, organ function, and disease status [7]. For relapsed or refractory ATL, multiagent chemotherapy can be reinitiated. Recently introduced agents such as mogamulizumab, an anti‐CC chemokine receptor 4 antibody, and lenalidomide, an immunomodulatory agent, are also used. By contrast, for patients with indolent ATL who do not have skin lesions, watchful waiting (in other words, monitoring without treatment) is recommended until acute transformation occurs.

It has been reported that approximately 50% of all patients with ATL, regardless of clinical subtype, have skin lesions [8, 9, 10, 11]. Eruptions specific to ATL vary widely; they are classified into the following types: patch, plaque, multipapular, erythrodermic, nodulotumoral, and purpuric.

The Japanese guidelines for the management of cutaneous lymphoma recommend topical steroids, ultraviolet (UV) irradiation, and radiotherapy as first‐line treatment for ATL with lesions that mainly involve the skin. Interferon (IFN), single‐agent chemotherapy, and retinoids are other potential therapies [11]. However, there is currently insufficient evidence that treatment of skin lesions improves the prognosis of indolent ATL. As a result, patients with indolent ATL have been managed with watchful waiting until disease progression [12]. However, the survival rate of the smoldering and chronic types is not good, and ATL‐specific eruption has been identified as an independent prognostic factor. Therefore, treatment of skin lesions with skin‐directed or systemic therapy (excluding cytotoxic anticancer agents) is expected to improve prognosis.

Bexarotene is a retinoid X receptor (RXR) agonist that binds selectively to the nuclear receptor RXR. Bexarotene was first approved in the United States in 1999 as an orphan drug for the indication of CTCL refractory to at least one systemic therapy. It was also approved in Japan for CTCL in 2016. Since bexarotene has been shown to be effective for CTCL [13, 14, 15], it was assumed that it would also be effective for ATL skin lesions. However, none of the participants in a previous clinical trial had ATL. Therefore, the safety, efficacy, and pharmacokinetics of bexarotene for ATL were not investigated. In addition, to our knowledge, there is only one case of bexarotene being used in patients with ATL in the USA [16, 17]. Thus, information on this drug for ATL is limited. This study was conducted to evaluate the pharmacokinetics and investigate the safety and efficacy of bexarotene in Japanese patients with ATL.

## 2 Methods

This study was a multicenter, open‐label, historically controlled, 2‐arm phase II study conducted between January 29, 2019, and May 24, 2022. Patients with ATL were enrolled in 12 hospitals in Japan. The safety, efficacy, and pharmacokinetics of bexarotene were assessed. All patients provided written informed consent. The study was conducted according to Good Clinical Practice guidelines and the Declaration of Helsinki. All protocols were approved by the institutional review board at each participating center. This study was registered with the Japan Registry of Clinical Trials (jRCT2080224172).

### 2.1 Patient Eligibility

Patients who were 20 years or older were included if they were confirmed as positive for serum anti‐HTLV‐1 antibody and diagnosed with ATL. Patients with aggressive ATL needed to have achieved response with one or more regimen(s) of systemic chemotherapy before participating in this clinical trial.

Chemotherapy, IFN preparations, radiotherapy, systemic steroids (except when continuously administered for diseases other than the primary disease at a prednisolone‐equivalent dose of 5 mg/day or less) were prohibited for 4 weeks; molecular‐targeted drugs for 12 weeks; UV light therapy for 3 weeks (except in patients whose conditions were stable after receiving treatment with long‐wavelength UV [UVA] or medium‐wavelength UV [UVB] for at least 12 weeks); topical corticosteroids for 2 weeks (except for corticosteroids that are “strong” or of lower strength); other retinoids, β‐carotene, and vitamin A for 4 weeks (except for dietary intake); and investigational drugs other than bexarotene for 12 weeks.

### 2.2 Study Design

This was an open‐label, multicenter, 2‐dose parallel‐group, phase II study to evaluate the safety, efficacy, and pharmacokinetics of bexarotene in patients with ATL skin lesions. For both the 100 and 300 mg/m^2^ initial dose groups randomized by allocation table to compare each group with the threshold response rate, the study consisted of a screening period within 2 weeks before starting bexarotene, a 24‐week treatment period, and a 4‐week follow‐up period. Patients took oral bexarotene once daily within 30 min after breakfast.

Safety was assessed according to the Common Terminology Criteria for Adverse Events version 4.0. If grade ≥ 3 drug‐related adverse events (AEs) or hypertriglyceridemia occurred during treatment, bexarotene was interrupted, reduced in dose, or terminated early in the clinical trial.

### 2.3 Assessments

Investigators measured the percentage of body surface area (BSA) affected by each lesion type (patch, plaque, or tumor) every 4 weeks. At the time of each measurement, the size of the patient's hand excluding the thumb was defined as 1% of BSA, and the size of the patient's palm excluding the fingers was defined as 0.5% of the BSA. A Modified Severity Weighted Assessment Tool (mSWAT) score was calculated on the basis of the percentage of BSA for each lesion (patch, plaque, and tumor) according to a previous report [18]. When the skin lesion was 100% resolved according to mSWAT, a skin biopsy was required to histopathologically confirm that there were no residual ATL tumor cells. If there were no residual tumor cells, the result was defined as a complete response (CR); otherwise, it was considered a partial response (PR). If skin lesions improved 50% or more, the response was also considered a PR. For mSWAT scores with a decrease of less than 50% to an increase of less than 25%, the response was stable disease (SD). If mSWAT score increased by 25% or greater, the response was progressive disease (PD).

A patch was defined as a lesion of any size without visible elevation or infiltration. It may be accompanied by dyschromia, scales, crusts, or folds. It should be noted that areas with purpura and erythema were also considered patches in this study.

A plaque was defined as a lesion of any size with elevation or infiltration. It may be accompanied by dyschromia, scaling, crusts, or follicular lesions. Papules were considered plaques in this study.

A tumor was defined as a single or nodular lesion ≥ 1 cm or an ulcerated plaque with deep or vertical growth.

Responses for ATL were assessed according to the partially modified response criteria for ATL [12] proposed by the International Consensus Meeting with some modifications (Table S1).

Laboratory tests (hematology, blood biochemistry, fasting lipid profile, thyroid function, and urinalysis), measurements of vital signs and body weight, 12‐lead electrocardiography (ECG), computed tomography (CT), and ophthalmologic slit‐lamp examinations were performed at baseline to gather information on safety variables. AEs, lipid profile, and thyroid function based on thyroid‐stimulating hormone and free thyroxine levels were evaluated weekly during the first 4 weeks, every 2 weeks until 8 weeks, and every 4 weeks thereafter. Hematology, blood biochemistry, and urinalysis were assessed every 2 weeks during the 4 weeks of treatment and every 4 weeks thereafter. Vital signs and body weight were measured every 4 weeks. ECG and CT were performed every 12 weeks. Slit‐lamp examination was performed every 24 weeks.

### 2.4 Endpoints

The primary efficacy endpoint was the objective response rate (ORR). ORR was defined as the proportion of patients whose best overall response was either CR or PR, as assessed with mSWAT. The secondary efficacy endpoints were the response rate (best overall response) assessed according to the modified response criteria for ATL; duration of response (DOR), time to response (TTR), disease control rate (DCR), and time‐to‐progression (TTP) according to partially modified ATL response criteria; and the proportion of patients with acute transformation of indolent ATL.

### 2.5 Statistical Analysis

The target sample size was calculated on the basis of the results of oral etoposide and prednisolone combination therapy (ETO therapy) [18]. From the lower limit of the 95% confidence interval (CI), the threshold response rate was assumed to be 15%, and the expected response rate was estimated to be 40% on the basis of the response rate for ETO therapy. For a statistical power of 80% and a 1‐sided *α* of 0.05, 19 patients were required in the 100 mg/m^2^/day and 300 mg/m^2^/day initial dose groups, respectively. The 95% confidence intervals (CIs) for response rates were calculated using the two‐sided Wilson's method. Median values of TTR, DOR, and TTP were estimated using the Kaplan–Meier product‐limit method, and the Brookmeyer and Crowley method was used to generate the 95% confidence interval for Kaplan–Meier median values.

Statistical analyses were conducted using SAS software, version 9.4 (SAS Institute). Continuous variables were summarized using medians and ranges. Categorical variables were described using frequencies and percentages. CIs were established using the Wilson method.

The full analysis set (FAS) population was defined as enrolled patients who received at least 1 dose of the study treatment. The safety analysis population was defined as patients in the FAS who had at least 1 data point for safety assessment after bexarotene administration. Notably, in this study, the safety analysis population corresponds to the FAS. The efficacy analysis population included patients in the FAS who were evaluated with mSWAT at least once.

## 3 Results

### 3.1 Patient Characteristics

**FIGURE 1:**
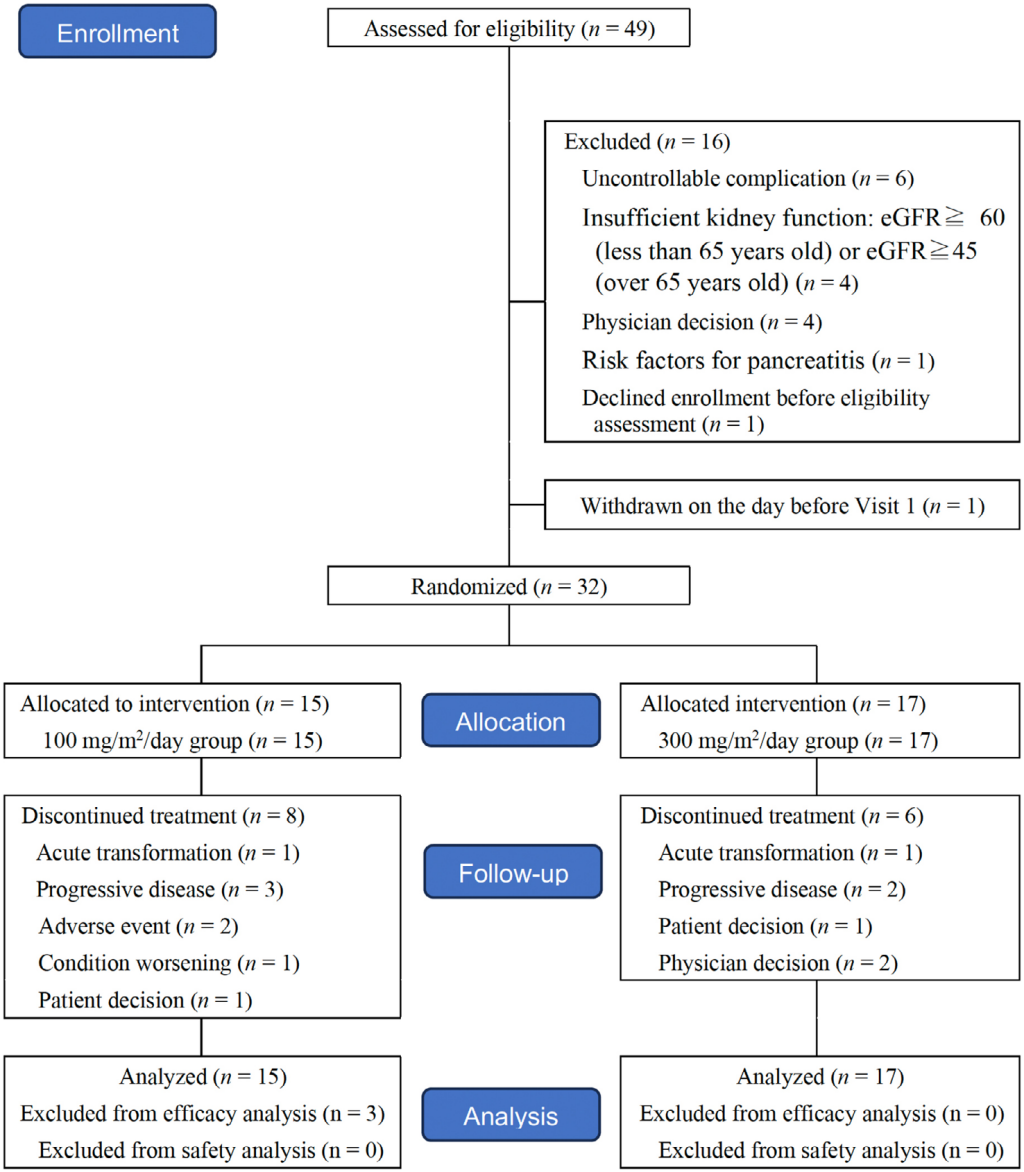
The enrollment flow diagram of this study.

Of 33 patients determined to be eligible for eligibility assessment, 32 patients were enrolled (Figure 1). One patient declined study participation after providing written informed consent but before the eligibility assessment.

The 100 mg/m^2^ initial dose group included 12 patients with smoldering type, 2 with favorable chronic type, and 1 with acute type. The 300 mg/m^2^ initial dose group consisted of 11 patients with smoldering type, 3 with favorable chronic type, and 3 with acute type (Table 1). In the 100 mg/m^2^ group, 7 of 15 (46.7%) patients completed the planned 24‐week treatment; in the 300 mg/m^2^ group, 11 of 17 (64.7%) completed the planned treatment. The remaining 8 (53.3%) patients in the 100 mg/m^2^ group discontinued treatment because of acute transformation (*n* = 1), PD with mSWAT evaluation (*n* = 2), PD with ATL response criteria (*n* = 1), AEs (*n* = 2), worsening condition (*n* = 1), or patient preference (*n* = 1). Six (35.3%) patients in the 300 mg/m^2^ initial dose group discontinued treatment for the following reasons: acute transformation (*n* = 1), PD with mSWAT evaluation (*n* = 1), PD with ATL response criteria (*n* = 1), patient preference (*n* = 1), and investigator's judgment (*n* = 2) (Table S2).

**TABLE 1:** Patient characteristics at baseline.

	100 mg/m^2^/day (*N* = 15)	300 mg/m^2^/day (*N* = 17)	Total (*N* = 32)
Age, years	71.0 (33–84)	66.0 (42–75)	68.0 (33–84)
Sex			
Male	7 (46.7)	7 (41.2)	14 (43.8)
Female	8 (53.3)	10 (58.8)	18 (56.3)
ATL subtype^a^			
Smoldering	12 (80.0)	11 (64.7)	23 (71.9)
Favorable chronic	2 (13.3)	3 (17.6)	5 (15.6)
Unfavorable chronic	0 (0.0)	0 (0.0)	0 (0.0)
Lymphoma	0 (0.0)	0 (0.0)	0 (0.0)
Acute	1 (6.7)	3 (17.6)	4 (12.5)
Prior treatment			
Topical steroids	12 (80.0)	14 (82.4)	26 (81.3)
Other topical medications	2 (13.3)	5 (29.4)	7 (21.9)
Oral retinoids	0 (0.0)	3 (17.6)	3 (9.4)
Other oral medications	1 (6.7)	1 (5.9)	2 (6.3)
Systemic chemotherapy	2 (13.3)	7 (41.2)	9 (28.1)
Ultraviolet irradiation	7 (46.7)	17 (100.0)	24 (75.0)
Radiotherapy	2 (13.3)	2 (11.8)	4 (12.5)
Allogenic hematopoietic stem cell transplantation	1 (6.7)	0 (0.0)	1 (3.1)
Surgical resection	2 (13.3)	1 (5.9)	3 (9.4)
Other investigational agent	0 (0.0)	1 (5.9)	1 (3.1)

^a^ ATL was divided into four subtypes according to the Shimoyama classification. Abbreviations: ATL, indicates adult T‐cell lymphoma/leukemia.

### 3.2 Efficacy

**FIGURE 2:**
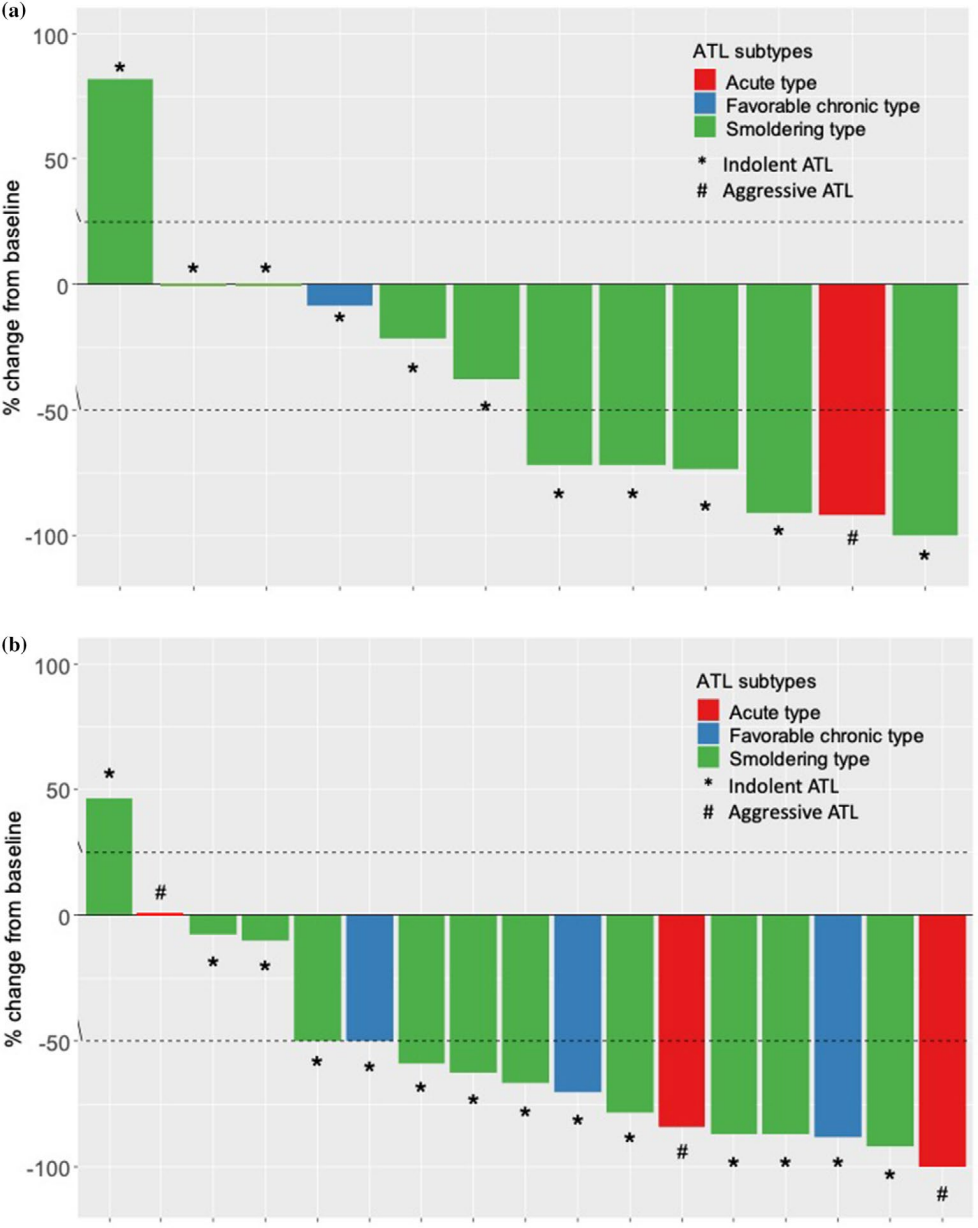
Best percentage change from baseline in modified Severity Weighted Assessment Tool (mSWAT) score of target lesions following treatment with bexarotene in 12 patients in the (a) 100 mg/m^2^ and (b) 300 mg/m^2^ initial dose groups. Best percentage change from baseline > 100%. 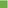: Smoldering type; 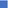: Favorable chronic type; 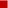: Acute type. *: indolent adult T‐cell lymphoma/leukemia (ATL); #: Aggressive ATL.

The primary analysis was conducted in an efficacy analysis population. The ORR (CR + PR) according to mSWAT is shown in Table 2 and Figure 2. Of 12 patients in the 100 mg/m^2^ initial dose group, 6 patients achieved CR or PR as the best overall response (ORR, 50.0%; 95% CI, 25.4%–74.6%, Figure 2a). Of 17 patients in the 300 mg/m^2^ initial dose group, 12 patients achieved CR or PR (ORR, 70.6%; 95% CI, 46.9%–86.7%, Figure 2b).

**TABLE 2:** Summary of clinical efficacy based on mSWAT score.

	*N*	Response rate (CR + PR), *n* (%) [95% CI]	CR	PR	SD	PD	NE
*100 mg/m* ^*2*^ */day*							
Total	12	6 (50.0%) [25.4–74.6]	0 (0.0)	6 (50.0)	3 (25.0)	1 (8.3)	2 (16.7)
Smoldering	10	5 (50.0%) [23.7–76.3]	0 (0.0)	5 (50.0)	2 (20.0)	1 (10.0)	2 (20.0)
Favorable chronic	1	0 (0.0%) [0.0–79.3]	0 (0.0)	0 (0.0)	1 (100.0)	0 (0.0)	0 (0.0)
Unfavorable chronic	0	—	—	—	—	—	—
Lymphoma	0	—	—	—	—	—	—
Acute	1	1 (100.0%) [20.7–100.0]	0 (0.0)	1 (100.0)	0 (0.0)	0 (0.0)	0 (0.0)
*300 mg/m* ^*2*^ */day*							
Total	17	12 (70.6%) [46.9–86.7]	0 (0.0)	12 (70.6)	3 (17.6)	1 (5.9)	1 (5.9)
Smoldering	11	8 (72.7%) [43.4–90.3]	0 (0.0)	8 (72.7)	2 (18.2)	1 (9.1)	0 (0.0)
Favorable chronic	3	2 (66.6%) [20.8–93.9]	0 (0.0)	2 (66.7)	0 (0.0)	0 (0.0)	1 (33.3)
Unfavorable chronic	0	—	—	—	—	—	—
Lymphoma	0	—	—	—	—	—	—
Acute	3	2 (66.7.%) [20.8–93.9]	0 (0.0)	2 (66.7)	1 (33.3)	0 (0.0)	0 (0.0)

*Note:* 95% CIs were calculated using Wilson's method.

Abbreviations: CI, confidence interval; CR, complete response; NE, not evaluable; PD, progressive disease; PR, partial response; SD, stable disease; mSWAT, modified Severity Weighted Assessment Tool.

There were no patients with CR according to mSWAT. In the 100 mg/m^2^ initial dose group, 5 of 10 patients (50.0%) with the smoldering type achieved PR, and 1 patient (100%) with the acute type achieved PR. In the 300 mg/m^2^ initial dose group, 8 of 11 patients (72.7%) with the smoldering type, 2 of 3 (66.7%) patients with the favorable chronic type, and 2 of 3 patients (66.7%) with the acute type achieved PR.

Patients with SD as the best overall response in the 100 mg/m^2^ initial dose group consisted of 2 of 10 (20.0%) patients with the smoldering type, 1 of 1 (100%) with the favorable chronic type, and 0 of 1 (0%) with the acute type. In the 300 mg/m^2^ initial dose group, SD was the best overall response in 2 of 11 patients (18.2%) with the smoldering type, 0 of 3 (0%) with the favorable chronic type, and 1 of 3 (33.3%) with the acute type.

**FIGURE 3:**
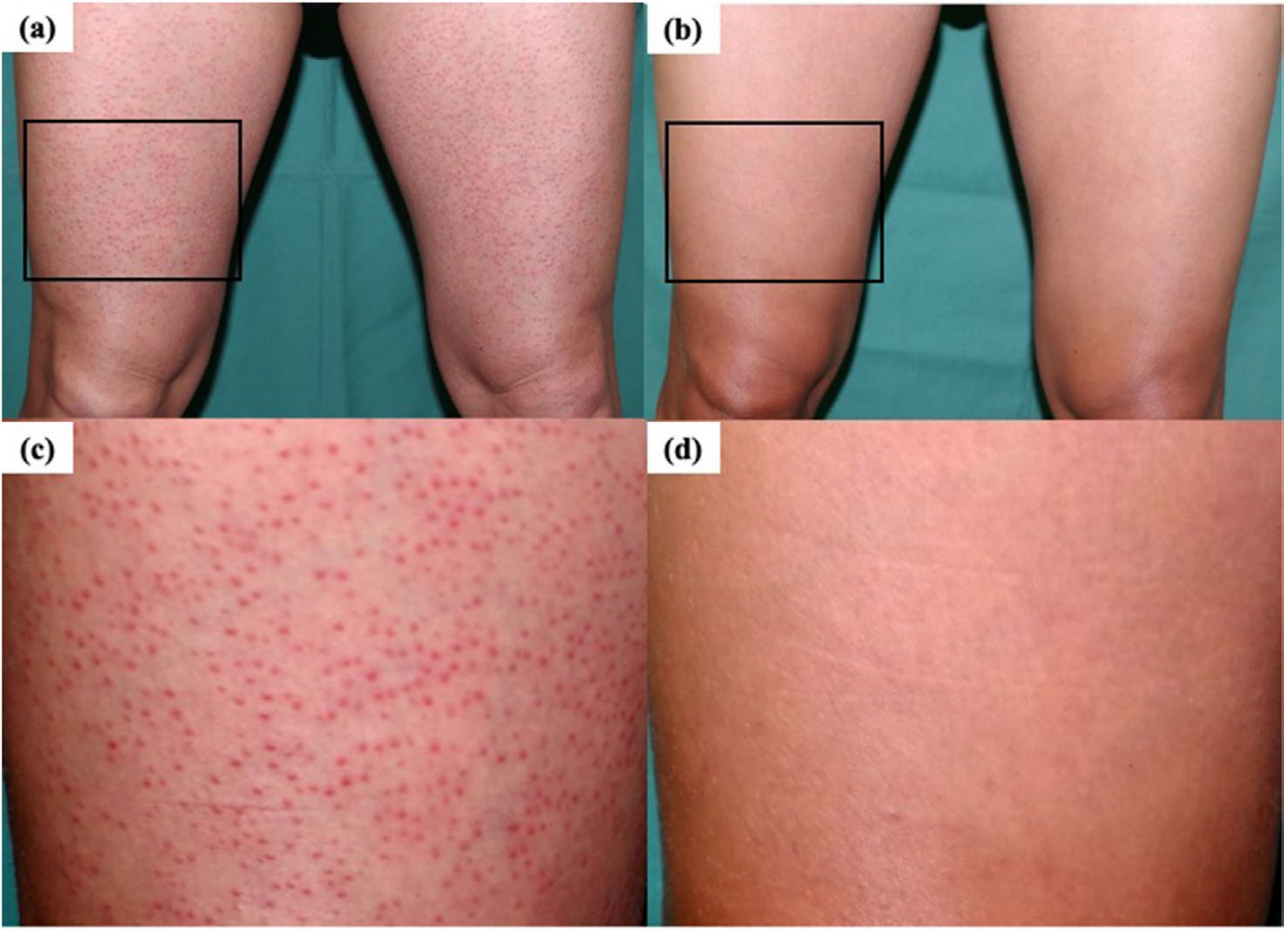
Photographs of a patient (a,c) before and (b,d) 24 weeks after bexarotene therapy. (a,c) mSWAT score, 108.0 (b,d) mSWAT score, 21.0.

PD was observed in 1 of 10 (10.0%) patients in the 100 mg/m^2^ and in 1 of 11 (9.1%) patients in the 300 mg/m^2^ with the smoldering type. Two of 10 (20.0%) patients with the smoldering type in the 100 mg/m^2^ initial dose group and 1 of 3 (33.3%) with the favorable chronic type in the 300 mg/m^2^ initial dose group were not evaluable (NE) because they had not been reexamined, and assessments were not finalized after an interval of at least 4 weeks. The representative features of mSWAT score improvement in one patient are shown in Figure 2. Multiple papules were observed in the patient's femoral region on Day 1 (Figure 3a,c); almost all of them disappeared after 24 weeks of treatment with bexarotene at an initial dose of 300 mg/m^2^ (Figure 3b,d).

The secondary efficacy assessment with modified response criteria for ATL was conducted with the efficacy analysis population. In the 100 mg/m^2^ initial dose group, 5 of 12 patients (41.7%; 95% CI, 19.3%–68.0%) had the best overall response of CR, complete response unconfirmed (CRu), or PR according to the modified response criteria for ATL. In the 300 mg/m^2^ initial dose group, 10 of 17 patients (58.8%; 95% CI, 36.0%–78.4%) had the best overall response of CR, CRu, or PR (Table 3).

**TABLE 3:** Efficacy with response evaluation criteria of ATL by the International Consensus Meeting with some modifications.

			100 mg/m^2^/day (*N* = 12)	300 mg/m^2^/day (*N* = 17)
	Response rate (CR + CRu + PR)	*n* (%) [95% CI]	5 (41.7) [19.3–68.0]	10 (58.8) [36.0–78.4]
	CR	*n* (%)	0 (0.0)	0 (0.0)
	CRu	*n* (%)	0 (0.0)	0 (0.0)
	PR	*n* (%)	5 (41.7)	10 (58.8)
	SD	*n* (%)	4 (33.3)	4 (23.5)
	PD	*n* (%)	3 (25.0)	3 (17.6)
	NE	*n* (%)	0 (0.0)	0 (0.0)
*Target lesion*				
Nodal/extranodal	Response rate (CR + CRu + PR)	*n*/*N* (%)	0/1 (0.0)	0/0 (0.0)
Skin	Response rate (CR + CRu + PR)	*n*/*N* (%)	6/12 (50.0)	12/17 (70.6)
Peripheral blood	Response rate (CR + CRu + PR)	*n*/*N* (%)	1/4 (25.0)	1/4 (25.0)
Bone marrow	Response rate (CR + CRu + PR)	*n*/*N* (%)	0/1 (0.0)	0/0 (0.0)

*Note:* None of the patients had liver or spleen invasion by ATL tumor cells.

Abbreviations: ATL indicates adult T‐cell lymphoma/leukemia; CI, confidence interval; CR, complete response; CRu, complete response unconfirmed; PD, progressive disease; PR, partial response; SD, stable disease; NE, not evaluable.

Only 1 patient in the 100 mg/m^2^ initial dose group had nodal or extranodal lesions (Table 3). It was difficult to determine whether bexarotene would have elicited a beneficial effect on these lesions. Four patients in each group had more than 5% ATL cells in the peripheral blood, of whom 1 patient in each group responded to bexarotene. One patient in the 100 mg/m^2^ initial dose group had bone marrow involvement at baseline. The response to bone marrow lesions was not assessed at any time point in this study.

None of the patients had ATL involvement in the liver or spleen.

DCR was 9 of 12 patients (75.0%; 95% CI, 46.8–91.1) in the 100 mg/m^2^ initial dose group and 14 of 17 (82.4%; 95% CI, 59.0–93.8) in the 300 mg/m^2^ initial dose group. Median TTR was 169 days (95% CI, 84‐NE) in the 100 mg/m^2^ initial dose group, and 85.0 days (95% CI, 85–NE) in the 300 mg/m^2^ initial dose group, respectively. Median DOR for each group was not reached. Median TTP for each group was not reached. The indolent ATL patients who underwent acute transformation in the study were 2 of 11 (18.2%; 95% CI, 5.1–47.7) in the 100 mg/m^2^ initial dose group and 1 of 14 (7.1%; 95% CI, 1.3–31.5) in the 300 mg/m^2^ initial dose group, respectively.

### 3.3 Pharmacokinetics

Serial and trough blood samples were collected for plasma bexarotene concentration measurements (Figure S1). The pharmacokinetic parameters after the first dose in the 100 mg/m^2^ initial dose group were as follows: maximum plasma concentration (*C*_max_), 890.8 ng/mL; area under the plasma concentration from 0 to 24 h (AUC_0–24_), 5695.8 ng·h/mL; and time to reach maximum plasma concentration (*t*_max_), 3.355 h. In the 300 mg/m^2^ initial dose group, they were as follows: *C*_max_, 2555 ng/mL; AUC_0–24_, 18 883 ng·h/mL; and *t*_max_, 3.400 h. During Day 15, the parameters in the 100 mg/m^2^ initial dose group were: *C*_max_, 631.1 ng/mL; AUC_0–24_, 3433.2 ng·h/mL; *t*_max_, 3.335 h; and *R*_AUC_, 0.7197. They were as follows in the 300 mg/m^2^ initial dose group: *C*_max_, 1518 ng/mL; AUC_0–24_, 10 725 ng·h/mL; *t*_max_, 3.260 h; and *R*_AUC_, 0.5938 (Table S3).

### 3.4 Safety

None of the study patients died during the study period. Three patients died outside of the study period; their deaths were attributed to worsening of the primary disease.

AEs occurred in all 15 patients (100%) in the 100 mg/m^2^ initial dose group and 16 of 17 patients (94.1%) in the 300 mg/m^2^ initial dose group. AEs with 20% or greater incidence are shown in Table 4. There were 3 (20.0%) patients in the 100 mg/m^2^ initial dose group and 8 (47.1%) in the 300 mg/m^2^ initial dose group who underwent dose adjustment or suspension caused by AEs.

**TABLE 4:** Treatment‐related adverse events in more than 20% of patients in either group.

System organ class^a^	100 mg/m^2^	300 mg/m^2^	Total
Preferred term^a^	(*N* = 15)	(*N* = 17)	(*N* = 32)
Any treatment‐related adverse event	15 (100)	16 (94.1)	31 (96.9)
Endocrine disorder	9 (60.0)	16 (94.1)	25 (78.1)
Hypothyroidism	9 (60.0)	15 (88.2)	24 (75.0)
Metabolism and nutrition disorder	10 (66.7)	15 (88.2)	25 (78.1)
Hypertriglyceridemia	9 (60.0)	8 (47.1)	17 (53.1)
Hypercholesterolemia	3 (20.0)	7 (41.2)	10 (31.2)
Dyslipidemia	0 (0.0)	5 (29.4)	5 (15.6)
Hepatobiliary disorder	0 (0.0)	6 (35.3)	6 (18.8)
Hepatic function abnormal	0 (0.0)	5 (29.4)	5 (15.6)

^a^ Adverse events terms were coded according to the Medical Dictionary for Drug Regulatory Activities.

Serious AEs, which are associated with a risk of death or hospitalization, were observed in 3 patients in the 100 mg/m^2^ initial dose group: condition aggravated, drug eruption, and Stevens–Johnson syndrome. Drug eruption and Stevens–Johnson syndrome resulted in discontinuation of treatment. None of the patients in the 300 mg/m^2^ initial dose group had serious AEs.

Grade 3 AEs in the 100 mg/m^2^ initial dose group consisted of neutropenia (*n* = 2, 13.3%), hyperkalemia (*n* = 1, 6.7%), hypertriglyceridemia (*n* = 1, 6.7%), drug eruption (*n* = 1, 6.7%), and condition aggravated (*n* = 1, 6.7%). In the 300 mg/m^2^ initial dose group, Grade 3 AEs consisted of leukopenia (*n* = 1, 5.9%), neutropenia (*n* = 2, 11.8%), hypertriglyceridemia (*n* = 2, 11.8%), dyslipidemia (*n* = 2, 11.8%), hepatic function disorder (*n* = 2, 11.8%), neutrophil count decreased (*n* = 2, 11.8%), and white blood cell count decreased (*n* = 1, 5.9%). One (6.7%) patient in the 100 mg/m^2^ initial dose group developed grade 4 Stevens–Johnson syndrome. Fourteen patients had grade ≥ 3 AEs; they recovered or their condition improved after 8–197 days of treatment suspension, except for 1 patient with hypertriglyceridemia in the 100 mg/m^2^ initial dose group and 1 patient with dyslipidemia in the 300 mg/m^2^ initial dose group. The condition of patients with these 2 AEs did not improve during the study period.

## 4 Discussion

This is the first report to assess the safety and efficacy of bexarotene in patients with ATL that was mainly focused on skin lesions. In both the 100 and 300 mg/m^2^ initial dose groups, the lower limit of the 95% CI of the response rate exceeded the threshold response rate of 15% defined for this study. Furthermore, we demonstrated that bexarotene is able to improve skin lesions associated with both indolent and aggressive ATL. In this clinical trial, none of the patients had CR assessed with mSWAT. This was due to the strict CR assessment criteria in mSWAT, which requires a skin biopsy to confirm the lack of residual lesions. Indeed, there was 1 patient with mSWAT score 0 at both initial doses; however, residual ATL tumor cells were confirmed via biopsy.

The bexarotene response rate in patients with ATL estimated with mSWAT was slightly higher than that in patients with CTCL [13, 14, 15]. Therefore, it could be possible that bexarotene is effective in improving the skin lesions in ATL of any type. There were a few patients who also had extracutaneous lesions. Bexarotene treatment markedly improved skin lesions, but not other lesions, in both initial dose groups. This implies that bexarotene selectively acts on skin lesions in patients with ATL. Nevertheless, we currently have no evidence about why bexarotene improved only skin lesions.

As in patients with CTCL [13], *C*_max_, AUC_0–24_, and *R*_AUC_ were lower on Day 15 compared with Day 1. Bexarotene is known to mildly induce and be metabolized by the drug‐metabolizing enzyme CYP3A [19]. Thus, it seems likely that repeated administration affects the metabolism of bexarotene via upregulation of CYP3A, leading to a decrease in the plasma concentration of bexarotene.

This clinical trial revealed that the safety profile of bexarotene in patients with ATL was similar to that in patients with CTCL. Grade ≥ 3 AEs included dyslipidemia, hypertriglyceridemia, leukopenia, neutropenia, neutrophil count decreased, white blood cell count decreased, and hepatic function disorder. Some patients experienced distinctive AEs. Hyperkalemia, drug eruption, and Stevens–Johnson syndrome, which were not observed in patients with CTCL, were only observed in the 100 mg/m^2^ initial dose group in this study [13, 14, 15]. Thus, it is conceivable that this event might have been caused by multiple factors including bexarotene administration, but the detailed reason remains unknown.

In this study, acute transformation was observed in 3 patients with indolent ATL: two in the 100 mg/m^2^ group and 1 in the 300 mg/m^2^ group. They were considered to be instances of ATL progression.

This study has several limitations. First, this was an open‐label study; the study had terminated with fewer than 38 patients who were originally planned. Second, this study selectively included patients with indolent and aggressive ATL who had stable or mild symptoms with performance status 0–2. Third, the evaluation of extracutaneous lesions was inadequate because the number of enrolled patients with extracutaneous lesions was small. Therefore, further study with data from real‐world practice is needed to investigate the safety and efficacy of combinations of bexarotene and other therapies.

Although highly effective drugs like mogamulizumab, lenalidomide, tucidinostat, and valemetostat have become available for the treatment of aggressive ATL, there are no treatment options for indolent ATL other than watchful waiting. In this study, bexarotene was clarified as a treatment for skin lesions in patients with indolent and aggressive ATL. Nevertheless, evidence remains limited, and further studies are needed to accumulate cases and compare the duration until acute transformation or progression‐free survival with historical controls.

The Revised ATL International Consensus Meeting Report published in 2019 recommended (outside of clinical trials) active monitoring or, if available, interferon alpha with zidovudine (AZT/IFN‐alpha) with or without arsenic trioxide and with or without topical therapies or phototherapy for symptomatic smoldering and favorable chronic ATL [6]. If AZT/IFN‐alpha is not available, treatment options are limited to skin‐directed therapy. AZT and IFN‐alpha are not approved for ATL in Japan. Bexarotene is available in more than 30 countries worldwide and in Japan; it is approved for the treatment of CTCL.

Taken together, our clinical data indicate that bexarotene could be useful as a new treatment specific to skin lesions.

## Conflicts of Interest

Author K.Y. has received honoraria from Meiji Seika Pharma, Kyowa Kirin, Daiichi Sankyo, Bristol Myers Squibb, Eisai, and Minophagen Pharmaceutical. T.S. received research funding from Sun Pharma. T.F. received honoraria and research funding from Minophagen Pharmaceutical. K.I. received honoraria from and has had advisory and consulting roles at Minophagen Pharmaceutical. I.M. received honoraria from Minophagen Pharmaceutical. The remaining authors declare no competing financial interests. T.I. is an Editorial Board member of Journal of Dermatology and a coauthor of this article. To minimize bias, they were excluded from all editorial decision‐making related to the acceptance of this article for publication. Takamichi Ito is an Editorial Board member of Journal of Dermatology and a coauthor of this article. To minimize bias, they were excluded from all editorial decision‐making related to the acceptance of this article for publication.

## Supporting information


**Figure S1.** Time course of plasma concentration after bexarotene administration on (A) Day 1 and (B) Day 15.
**Table S1a.** Response criteria for adult T‐cell lymphoma/leukemia (ATL) proposed by the International Consensus Meeting with some odification.
**Table S1b.** Modified response criteria for adult T‐cell lymphoma/leukemia (ATL).
**Table S2.** Details of the reason why the investigator decided that the patient should withdraw from the trial.
**Table S3.** Summary of pharmacokinetics parameters after bexarotene administration on Days 1 and 15.

## Data Availability

Data are available on request from the corresponding author, Kentaro Yonekura (ke.yonekura@jiaikai.jp).
