# Supplementary material for: An Open‐Label, Multicenter, Phase II Study of Bexarotene in Patients with Adult T‐Cell Leukemia/Lymphoma
Source: J Dermatol. 2025 Aug 23;52(11):1619–28. doi: 10.1111/1346-8138.17919 (PMC12592592; doi:10.1111/1346-8138.17919)
Supplement: Supplementary file 1 — Figure S1. Time course of plasma concentration after bexarotene administration on (A) Day 1 and (B) Day 15. Table S1a. Response criteria for adult T‐cell lymphoma/leukemia (ATL) proposed by the International Consensus Meeting with some odification. Table S1b. Modified response criteria for adult T‐cell lymphoma/leukemia (ATL). Table S2. Details of the reason why the investigator decided that the patient should withdraw from the trial. Table S3. Summary of pharmacokinetics parameters after bexarotene administration on Days 1 and 15. [file JDE-52-1619-s001.docx]

Supplemental Methods

Pharmacokinetics

Pharmacokinetic (PK) analysis was conducted using Phoenix WinNonlin, version 8.2 or later (Certara, Radnor, PA, USA), and SAS, version 9.4 (SAS Institute, Cary, NC, USA). Patient blood samples were collected at protocol-defined time points for PK analysis. PK parameters were calculated using noncompartmental analysis of plasma concentration–time data for bexarotene, including maximum plasma concentration (C_max_), time to reach maximum plasma concentration (T_max_), area under the plasma concentration-time curve from 0 to 24 hours (AUC_0-24_), area under the plasma concentration-time curve from time 0 to infinity terminal half-life(t_1/2_), elimination rate constant ((λz), and accumulation ratio (R_AUC_)

**Supplementary Figure 1**. **Time course of plasma concentration after bexarotene administration on (A) day 1 and (B) day 15**

**(A)**

† N=14 for 24 hours

‡ N=15 for 24 hours

**(B)**

† N=11 for 24 hours

‡ N=10 for 24 hours

Supplementary Table 1a Response criteria for adult T-cell lymphoma/leukemia (ATL) proposed by the International Consensus Meeting with some modification

| Response | Definition | Lymph Nodes | Extranodal Masses | Spleen, Liver | Skin | Peripheral Blood | Bone Marrow |
| --- | --- | --- | --- | --- | --- | --- | --- |
| Complete remission | Disappearance of all disease | Normal | Normal | Normal | Normal | Normal | Normal |
| Uncertified complete remission | Stable residual mass in bulky lesion | ≥ 75% decrease | ≥ 75% decrease | Normal | Normal | Normal | Normal |
| Partial remission | Regression of disease | ≥ 50% decrease | ≥ 50% decrease | No increase | ≥ 50% decrease | ≥ 50% decrease | Irrelevant |
| Stable disease | Failure to attain complete/partial remission and no progressive disease | No change in size | No change in size | No change in size | No change in size | No change | No change |
| Relapsed disease or progressive disease | New or increased lesions | New or ≥ 50% increase | New or ≥ 50% increase | New or ≥ 50% increase | ≥ 50% increase | New or ≥ 50% increase^‖^ | Reappearance |
| Not assessable |  |  |  |  |  |  |  |

Supplementary Table 1b Modified response criteria for adult T-cell lymphoma/leukemia (ATL)

| Response | SPD of Target Lesion  (If patients had any target lesion) | Non Target Lymph Nodes | Non Target  Extranodal Masses | Spleen, Liver | Skin  (mSWAT) | Peripheral Blood | Bone Marrow | New Lesion |
| --- | --- | --- | --- | --- | --- | --- | --- | --- |
| Complete remission (CR) | 100% disappearance | Normal | Normal | Normal | CR | Normal | Negative | No |
| Uncertified complete remission (CRu) | ≥75% decrease | Normal | Normal | Normal | CR | Normal | Negative | No |
| Partial remission (PR) | ≥50% decrease | Normal or No increase | Normal or  No increase | Normal or  No increase | PR | ≥ 50% decrease | Irrelevant | No |
| Stable disease (SD) | <50% decrease and  <50% increase | Normal or No increase | Normal or  No increase | Normal or  NJo increase | SD | No change | Irrelevant | No |
| progressive disease (PD) | ≥50% increase | Increase | Increase | Progression | PD | ≥ 50% increase | Reappearance | Yes |

Supplementary Table 2 Details of the reason why the investigator decided that the patient should withdraw from the trial.

| Patient No. | Details of investigator’s judgment |
| --- | --- |
| Patient 1 | The evaluation of the mSWAT and the ATL response criteria did not categorized the case as PD, and the patient had no symptoms of acute transformation. However, the patient complained of the pain in the skin lesion, and the investigator determined that treatment should be prioritize, leading to the discontinuation of the trial. |
| Patient 2 | Although the mSWAT assessment categorized the case as SD, the nodular lesions showed the tendency to increase. Taking everything into account, the investigator determined that the patient should discontinue the trial. |

# Supplementary Table 3. Summary of pharmacokinetics parameters after bexarotene administration on days 1 and 15

|  |  |  | Pharmacokinetic parameters in plasma | | | | | | | |
| --- | --- | --- | --- | --- | --- | --- | --- | --- | --- | --- |
| Initial dose |  | Statistic | C_max_ | AUC_0-24_ | AUC_inf_ | t_max_ | 𝛌z | t_1/2_ | R_Cmax_ | R_AUC_ |
|  |  |  | (ng/mL) | (ng·h/mL) | (ng·h/mL) | (h) | (/h) | (h) |  |  |
| 100 mg/m^2^ | Day 1 | N | 15 | 12 | 12 | 15 | 12 | 12 | N/A | N/A |
| (N=15) |  | Mean | 890.8 | 5695.8 | 5566.9 | 3.355 | 0.27685 | 2.655 | N/A | N/A |
|  |  | SD | 363.0 | 3564.0 | 3675.0 | 1.002 | 0.082987 | 0.564 | N/A | N/A |
|  |  | Median | 804.0 | 4898.5 | 4547.6 | 4.000 | 0.24628 | 2.815 | N/A | N/A |
|  |  | Minimum | 514 | 2061 | 1866 | 1.92 | 0.2127 | 1.44 | N/A | N/A |
|  |  | Maximum | 1920 | 15960 | 16090 | 4.17 | 0.4811 | 3.26 | N/A | N/A |
| 100 mg/m^2^ | Day 15 | N | 12 | 10 | 10 | 12 | 10 | 10 | 12 | 7 |
| (N=15) |  | Mean | 631.1 | 3433.2 | 3449.6 | 3.335 | 0.22416 | 3.516 | 0.7961 | 0.7197 |
|  |  | SD | 261.5 | 1402.5 | 1423.5 | 0.994 | 0.090419 | 1.389 | 0.3048 | 0.2224 |
|  |  | Median | 625.5 | 3176.6 | 3286.5 | 3.935 | 0.21150 | 3.290 | 0.7824 | 0.6917 |
|  |  | Minimum | 201 | 2266 | 2033 | 1.90 | 0.1004 | 1.54 | 0.293 | 0.428 |
|  |  | Maximum | 1260 | 6988 | 7007 | 4.18 | 0.4489 | 6.90 | 1.296 | 1.099 |
| 300 mg/m^2^ | Day 1 | N | 17 | 14 | 14 | 17 | 14 | 14 | N/A | N/A |
| (N=17) |  | Mean | 2555 | 18883 | 19039 | 3.400 | 0.22354 | 3.294 | N/A | N/A |
|  |  | SD | 655.4 | 5802.2 | 5832.6 | 1.953 | 0.057920 | 0.817 | N/A | N/A |
|  |  | Median | 2620 | 20044 | 20117 | 3.830 | 0.20836 | 3.328 | N/A | N/A |
|  |  | Minimum | 1220 | 6513 | 6523 | 0.98 | 0.1496 | 2.17 | N/A | N/A |
|  |  | Maximum | 3440 | 27190 | 27430 | 8.17 | 0.3197 | 4.63 | N/A | N/A |
| 300 mg/m^2^ | Day 15 | N | 13 | 12 | 12 | 13 | 12 | 12 | 13 | 12 |
| (N=17) |  | Mean | 1518 | 10725 | 10981 | 3.260 | 0.16533 | 5.134 | 0.5668 | 0.5938 |
|  |  | SD | 875.7 | 6197.5 | 6123.7 | 1.821 | 0.055014 | 3.582 | 0.2535 | 0.2682 |
|  |  | Median | 1340 | 10669 | 10869 | 3.850 | 0.16026 | 4.325 | 0.5801 | 0.6715 |
|  |  | Minimum | 491 | 2251 | 3084 | 0.97 | 0.04316 | 2.81 | 0.156 | 0.130 |
|  |  | Maximum | 3020 | 20870 | 20950 | 8.00 | 0.2468 | 16.06 | 0.941 | 0.970 |

AUC_0-24_: indicates area under the plasma concentration time 0 to 24 hours; AUC_inf_, area under the plasma concentration-time curve from time 0 to infinity; C_max_, maximum concentration; N: number of patients; N/A, not applicable; λz, elimination rate constant; t_1/2_, half-life; t_max_, time to maximum concentration; R_cmax_, ratio of C_max_ on day 1 to C_max_ on day 15; R_AUC_, ratio of AUC_0–24_ on day 1 to AUC_0-24_ on day 15; SD, standard deviation

Ns of AUC_0–24_, AUC_inf_ and t_1/2_ are equal to N of 𝛌z because they have been calculated using λz, whereas N of λz did not correspond to N of C_max_ or t_max_ both of which are independent of λz.
R_Cmax_ and R_AUC_ were calculated using data from patients with data available from days 1 and 15.
